# A New Methodology of Spatial Cross-Correlation Analysis

**DOI:** 10.1371/journal.pone.0126158

**Published:** 2015-05-19

**Authors:** Yanguang Chen

**Affiliations:** Department of Geography, College of Urban and Environmental Sciences, Peking University, 100871, Beijing, China; University of Rijeka, CROATIA

## Abstract

Spatial correlation modeling comprises both spatial autocorrelation and spatial cross-correlation processes. The spatial autocorrelation theory has been well-developed. It is necessary to advance the method of spatial cross-correlation analysis to supplement the autocorrelation analysis. This paper presents a set of models and analytical procedures for spatial cross-correlation analysis. By analogy with Moran’s index newly expressed in a spatial quadratic form, a theoretical framework is derived for geographical cross-correlation modeling. First, two sets of spatial cross-correlation coefficients are defined, including a global spatial cross-correlation coefficient and local spatial cross-correlation coefficients. Second, a pair of scatterplots of spatial cross-correlation is proposed, and the plots can be used to visually reveal the causality behind spatial systems. Based on the global cross-correlation coefficient, Pearson’s correlation coefficient can be decomposed into two parts: direct correlation (partial correlation) and indirect correlation (spatial cross-correlation). As an example, the methodology is applied to the relationships between China’s urbanization and economic development to illustrate how to model spatial cross-correlation phenomena. This study is an introduction to developing the theory of spatial cross-correlation, and future geographical spatial analysis might benefit from these models and indexes.

## Introduction

In geographical research, spatial correlation processes falls into two types: autocorrelation and cross-correlation. The former reflects intra-sample correlation, that is, a relationship between one measure and itself, while the latter reflects inter-sample correlation, namely, a relationship between one measure and another measure. Spatial autocorrelation is defined by one size measurement (e.g. city population) and one spatial contiguity measurement (e.g., Euclidean distance), while spatial cross-correlation can be defined by two size measurements (e.g., city population and urban area) and one spatial contiguity measurement. Based on the statistical measurements of Moran’s index and Geary’s coefficient [1, 2], a relatively mature theory has been developed for spatial autocorrelation analysis [3–25]. Spatial autocorrelation modeling has been widely applied to various correlational analyses of natural and human phenomena in many fields [26–41], and in particular it has been integrated into the spatial analytical technology of geographical information systems (GIS) [42, 43]. In contrast, the theory and methodology of spatial cross-correlation has not yet been well constructed for geographical analysis, despite the concept of “spatial cross-correlation” emerging in literature [44–51].

Mathematical modeling has been baffled for a long time by two factors: one is time lag, and the other, spatial dimension [52]. Where there is a time lag, there is a nonlinear process. On the other, where there is a spatial variable, there is a dimensional dilemma. Cross-correlation functions can be employed to solve the problems caused by time delay effect and thus to reveal the dynamic causality, but the correlational modeling of time series was obstructed by nonstationarity and fluctuation. The method of detrended cross-correlation analysis (DCCA) can be utilized to treat the nonstationary cross-correlation processes [53], and the random matrix theory (RMT) can be adopted to analyze the cross-correlation with fluctuation [54]. Because of DCCA, RMT, and fractal theory, the studies on temporal cross-correlation analysis develop rapidly in recent years [45, 55–69]. In a correlation function, if the time lag parameter is replaced by a spatial displacement parameter, a temporal correlation model will become a 1-dimensional spatial correlation model [70]; if the spatial displacement parameter is further substituted by a spatial weight function, the 1-dimensional spatial correlation model will change to a 2-dimensional spatial correlation model [7]. Fractal geometry has been used to develop spatial correlation modeling because a fractal model is always a correlation function [71, 72]. In theory, the temporal cross-correlation can be associated with spatial cross-correlation by the ideas from allometry, fractals, and hierarchy [73]. Before doing so, it is necessary to develop a methodology of spatial cross-correlation analysis.

The conditions of establishing spatial cross-correlation theory are ripe now, and it is time to solve many problems of spatial dimension in geographical analysis. For a number of elements within a regional system, the relationship between two measurements used to be characterized with Pearson’s correlation coefficient, which indicates the simplest cross-correlation. However, Pearson’s correlation coefficient shows nothing about interactions based on spatial distances. In this paper, a new theoretical framework for spatial correlation analysis is proposed for geographical research. The novelty of this framework rests with three aspects. First, it is founded on the analogy of a new expression of Moran’s index [7], which is similar in form to the random correlation matrix [63]. Therefore, the definition of spatial cross-correlation coefficient is easy to understand, and the relationship between spatial autocorrelation and spatial cross-correlation is clear. Second, it is formulated in the simplest form of vectors and matrices, so it is easy to calculate the cross-correlation coefficients and the related parameters. Third, the procedure of calculations and analysis is well developed on the basis of models, algorithms, and statistic tests. The methodology contains a set of measurements and graphs such as global indices, local indices, and cross-correlation scatterplots.

The rest of the article is arranged as follows. In Section 2 (*Results*), the global and local indices of spatial cross-correlation are defined by means of mathematical derivation and reasoning, and a pair of spatial cross-correlation scatterplots is presented by analogy with Moran’s scatterplots. In Section 3 (*Discussion*), based on the idea from spatial cross-correlation, Pearson’s correlation coefficient is decomposed into two parts: a direct correlation coefficient and an indirect correlation coefficient. A comparison is drawn between the spatial cross-correlation coefficient and Moran’s autocorrelation index. In Section 4 (*Materials and Methods*), as a case study, the analytical process of spatial cross-correlation is applied to the system of China’s cities and regions to research the relationships between urbanization and economic development. Finally, the paper concludes with a summary of the main points of this work.

## Results

### Global and local measurements of spatial cross-correlation

The theoretical framework of spatial cross-correlation analysis consists of a set of models and measurements. The basic mathematical reasoning is helpful for understanding these models and indices. Suppose there are *n* elements (e.g., cities) in a system (e.g., a network of cities and its hinterland) which can be measured by two variables (e.g., city population and urban area), *X* and *Y*. A pair of vectors can be defined as below:

$$X={\left[\begin{array}{cccc} x_{1} & x_{2} & \cdots & x_{n} \end{array}\right]}^{\text{T}},\;Y={\left[\begin{array}{cccc} y_{1} & y_{2} & \cdots & y_{n} \end{array}\right]}^{\text{T}}\;,$$ (1)

where *x*_*i*_ and *y*_*i*_ are two size measurements of the *i*th element (*i* = 1, 2, …, *n*), and the symbol “T” denotes transpose. The centralized variables can be calculated by

$$X_{\text{c}}=X-\mu_{x}\;,\;Y_{\text{c}}=Y-\mu_{y}\;,$$ (2)

where *μ*_*x*_ and *μ*_*y*_ represent the average values of the variables *x*_*i*_ and *y*_*i*_, which are expressed as

$$\mu_{x}=\frac{1}{n}\sum_{i=1}^{n}x_{i}\;,\;\mu_{y}=\frac{1}{n}\sum_{i=1}^{n}y_{i}\;.$$ (3)

The population variances of the two variables are as follows

$$\sigma_{x}^{2}=\frac{1}{n}\sum_{i=1}^{n}{\left(x_{i}-\mu_{x}\right)}^{2}=\frac{1}{n}X_{\text{c}}^{\text{T}}X_{\text{c}}\;,\;\sigma_{y}^{2}=\frac{1}{n}\sum_{i=1}^{n}{\left(y_{i}-\mu_{y}\right)}^{2}=\frac{1}{n}Y_{\text{c}}^{\text{T}}Y_{\text{c}}\;,$$ (4)

where *σ*_*x*_ and *σ*_*y*_ denote the population standard deviations of *x*_*i*_ and *y*_*i*_, respectively. The results of a scaling transform of the centralized variables form a pair of standardized vectors such as

$$x=\frac{X-\mu_{x}}{\sigma_{x}}=\frac{X_{\text{c}}}{\sigma_{x}}\;,\;y=\frac{Y-\mu_{y}}{\sigma_{y}}=\frac{Y_{\text{c}}}{\sigma_{y}}\;,$$ (5)

which are termed *standard scores* in statistics. It can be shown that the norm of *x* and *y*, i.e., the lengths of the two vectors, ‖*x*‖ and ‖*y*‖, exactly equals the dimensions of the vectors, i.e., the number of elements in the system, *n*. Thus we have

$$\left\|x\right\|=x^{\text{T}}x=n\;,\;\left\|y\right\|=y^{\text{T}}y=n\;.$$ (6)

The models of spatial correlation, including autocorrelation and cross-correlation, are based on spatial distance or spatial contiguity. Define an *n*-by-*n* unitary spatial weights matrix such as

$$W={\left[w_{ij}\right]}_{n\times n},$$ (7)

which is actually a unitized spatial weights matrix (USWM). The matrix can be produced by a spatial contiguity matrix (SCM), and it has three properties as below: (1) Symmetry, i.e., *w*_*ij*_ = *w*_*ji*_; (2) Zero diagonal elements, namely, **|** *w*_*ii*_ **|** = 0, which implies that the entries in the diagonal are all 0; (3) Unitization condition, that is

$$\sum_{i=1}^{n}\sum_{j=1}^{n}w_{ij}=1.$$ (8)

Then, by analogy with the improved formula of Moran’s index for spatial autocorrelation (Chen, 2013a), a new measurement for spatial cross-correlation analysis can be defined as

$$R_{\text{c}}=x^{\text{T}}Wy,$$ (9)

where *R*_c_ denotes the coefficient of spatial cross-correlation, which can be termed *spatial cross-correlation index* (SCI). It is easy to prove that the SCI is a correlation coefficient, and its value falls between -1 and 1. Because of symmetry of the spatial weights matrix, transposing *R*_c_ yields another expression

$$R_{\text{c}}={\left(x^{\text{T}}Wy\right)}^{\text{T}}=y^{\text{T}}W^{\text{T}}x=y^{\text{T}}Wx,$$ (10)

which is numerically equivalent to Eq (9). However, as indicated in the following section, from Eqs (9) and (10), we can derive different models for different uses of spatial analysis.

A set of matrix equations can be constructed on the basis of the SCI formulae. Eqs (9) and (10) multiplied left by *x* or *y* on both sides of the equal signs yields

$$M^{\left(xy\right)}x=xy^{\text{T}}Wx=R_{\text{c}}x,$$ (11)

$$M^{\left(yx\right)}y=yx^{\text{T}}Wy=R_{\text{c}}y,$$ (12)

$$M^{\left(xx\right)}y=xx^{\text{T}}Wy=R_{\text{c}}x,$$ (13)

$$M^{\left(yy\right)}x=yy^{\text{T}}Wx=R_{\text{c}}y.$$ (14)

It is easy to demonstrate that *xy* ^T^ *Wx* = *xx* ^T^ *Wy*, *yx* ^T^ *Wy* = *yy* ^T^ *Wx*. In these equations, there are two ideal spatial correlation matrixes (ISCM) for spatial autocorrelation as below:

$$M^{\left(xx\right)}=xx^{\text{T}}W\;,\;M^{\left(yy\right)}=yy^{\text{T}}W.$$ (15)

There are two ISCMs for spatial cross-correlation such as

$$M^{\left(xy\right)}=xy^{\text{T}}W,\;M^{\left(yx\right)}=yx^{\text{T}}W.$$ (16)

Eqs (11) and (12) show that SCI is just the eigenvalues of the ISCMs of spatial cross-correlation. This differs from Moran’s index, which is the characteristic value of the ISCM of spatial autocorrelation [7].

An important measurement of spatial autocorrelation is called local indicators of spatial association (LISA). LISA is also termed local Moran’s index [3]. Similarly, two sets of local spatial cross-correlation coefficients can be defined by

$$R_{i}^{\left(xy\right)}=x_{i}\sum_{j=1}^{n}w_{ij}y_{j}\;,$$ (17)

$$R_{j}^{\left(yx\right)}=y_{i}\sum_{j=1}^{n}w_{ij}x_{j}\;,$$ (18)

where *R*_*i*_ and *R*_*j*_ refer to the *local spatial cross-correlation index* (LSCI) of the *i*th element and the *j*th element. Accordingly, *R*_c_ denotes the *global spatial cross-correlation index* (GSCI), which can be termed SCI for short. The GSCI is used to reflect the summation of cross-correlation between any two elements, while the LSCI is utilized to measure the cross-correlation between a given element and all other elements in a geographical system. As *w*_*ij*_ = *w*_*ji*_, for arbitrary *n*, LSCI can be expressed with matrix equations as follows

$$\left[\begin{array}{c} x_{1}\\ x_{2}\\ \vdots \\ x_{n} \end{array}\right]\left[\begin{array}{cccc} y_{1} & y_{2} & \cdots & y_{n} \end{array}\right]\left[\begin{array}{cccc} w_{11} & w_{12} & \cdots & w_{1n}\\ w_{21} & w_{22} & \cdots & w_{2n}\\ \vdots & \vdots & \ddots & \vdots \\ w_{n1} & w_{n2} & \cdots & w_{nn} \end{array}\right]=\left[\begin{array}{cccc} x_{1}\sum_{j=1}^{n}w_{1j}y_{j} & x_{1}\sum_{j=1}^{n}w_{2j}y_{j} & \cdots & x_{1}\sum_{j=1}^{n}w_{nj}y_{j}\\ x_{2}\sum_{j=1}^{n}w_{1j}y_{j} & x_{2}\sum_{j=1}^{n}w_{2j}y_{j} & \cdots & x_{2}\sum_{j=1}^{n}w_{nj}y_{j}\\ \vdots & \vdots & \ddots & \vdots \\ x_{n}\sum_{j=1}^{n}w_{1j}y_{j} & x_{n}\sum_{j=1}^{n}w_{2j}y_{j} & \cdots & x_{n}\sum_{j=1}^{n}w_{nj}y_{j} \end{array}\right],$$ (19)

$$\left[\begin{array}{c} y_{1}\\ y_{2}\\ \vdots \\ y_{n} \end{array}\right]\left[\begin{array}{cccc} x_{1} & x_{2} & \cdots & x_{n} \end{array}\right]\left[\begin{array}{cccc} w_{11} & w_{12} & \cdots & w_{1n}\\ w_{21} & w_{22} & \cdots & w_{2n}\\ \vdots & \vdots & \ddots & \vdots \\ w_{n1} & w_{n2} & \cdots & w_{nn} \end{array}\right]=\left[\begin{array}{cccc} y_{1}\sum_{j=1}^{n}w_{1j}x_{j} & y_{1}\sum_{j=1}^{n}w_{2j}x_{j} & \cdots & y_{1}\sum_{j=1}^{n}w_{nj}x_{j}\\ y_{2}\sum_{j=1}^{n}w_{1j}x_{j} & y_{2}\sum_{j=1}^{n}w_{2j}x_{j} & \cdots & y_{2}\sum_{j=1}^{n}w_{nj}x_{j}\\ \vdots & \vdots & \ddots & \vdots \\ y_{n}\sum_{j=1}^{n}w_{1j}x_{j} & y_{n}\sum_{j=1}^{n}w_{2j}x_{j} & \cdots & y_{n}\sum_{j=1}^{n}w_{nj}x_{j} \end{array}\right],$$ (20)

which provide a simple approach to calculate LSCIs. Comparing Eqs (19) and (20) with Eqs (17) and (18) shows that the elements in the diagonals of *M* ^(*xy*)^ and *M* ^(*yx*)^ give the LSCI values. The traces of *M* ^(*xy*)^ or *M* ^(*yx*)^ are equal to the GSCI value. It is convenient for us to compute the LSCIs by means of matrix operations based on Eqs (19) and (20).

### Practical equations for spatial cross-correlation

In practice, the spatial cross-correlation coefficient can be defined in another form. The precondition for Eq (9) is as follows

$$nWy=R_{\text{c}}x,$$ (21)

which represents a practical relation for SCI. According to Eq (6), Eq (21) multiplied left by *x* ^T^ yields *nx* ^T^ *Wy* = *x* ^T^ *R*_c_ *x* = *nR*_c_, which results in Eq (9). Similarly, the precondition Eq (10) is as below

$$nWx=R_{\text{c}}y,$$ (22)

which multiplied left by *y* ^T^ yields *ny* ^T^ *Wx* = *y* ^T^ *R*_c_ *y* = *nR*_c_, and thus yields Eq (10). A real spatial correlation matrix (RSCM) for cross-correlation can be defined as

$$M=nW=\left\|x\right\|W=\left\|y\right\|W=x^{\text{T}}xW=y^{\text{T}}yW.$$ (23)

It can be proved that *R*_c_ is just the eigenvalue of *M*, and the corresponding eigenvector is (*x*+*y*). Actually, Eq (21) plus Eq (22) yields

$$M(x+y)=nW\;(x+y)=R_{\text{c}}(x+y).$$ (24)

This suggests that *M* corresponds to *M* ^(*xy*)^ and *M* ^(*yx*)^. The relationship between Eq (13) and Eq (21) gives an error equation

$$(M^{\left(xx\right)}-M)y=(xx^{\text{T}}W-nW)y=U,$$ (25)

in which *U* represents an error vector. The relationship between Eq (14) and Eq (22) gives another error equation

$$(M^{\left(yy\right)}-M)x=(yy^{\text{T}}W-nW)x=V,$$ (26)

in which *V* represents another error vector. There are errors between *My* = *x* ^T^ *xWy* and *M* ^(*xx*)^ *y* = *xx* ^T^ *Wy*, also there are errors between *Mx* = *y* ^T^ *yWx* and *M* ^(*yy*)^ *x* = *yy* ^T^ *Wx*. This suggests an approach to testing the “goodness of fit” of a spatial cross-correlation model relative to observational data. If the spatial cross-correlation is strong, *Mx* will be a close to *M* ^(*yy*)^ *x*, and *My* will be a close to *M* ^(*xx*)^ *y*.

### Spatial cross-correlation scatterplots

Spatial cross-correlation can be visually displayed with two scatterplots, which are similar to Moran’s scatterplot of spatial autocorrelation. However, the cross-correlation scatterplots come in pairs. In order to create the scatterplots, six variables based on the spatial correlation matrix are defined as below:

$$f^{\left(xy\right)}=M^{\left(xy\right)}x=xy^{\text{T}}Wx,$$ (27)

$$f^{\left(yx\right)}=M^{\left(yx\right)}y=yx^{\text{T}}Wy,$$ (28)

$$f^{\left(xx\right)}=M^{\left(xx\right)}y=xx^{\text{T}}Wy,$$ (29)

$$f^{\left(yy\right)}=M^{\left(yy\right)}x=yy^{\text{T}}Wx.$$ (30)

$$f^{\left(x\right)}=Mx=nWx.$$ (31)

$$f^{\left(y\right)}=My=nWy.$$ (32)

Using these equations, we can generate a set of scatterplots comprising four graphs with observational data and the corresponding calculations.

The newly defined variables can be matched to make cross-correlation scatterplots as follows. The relationship between *x* and *f* ^(*xy*)^ give the first scatterplot, the relationship between *x* and *f* ^(*xx*)^ give the second scatterplot, the relationship between *y* and *f* ^(*yx*)^ give the third scatterplot, and the relationship between *y* and *f* ^(*yy*)^ give the fourth scatterplot (Table 1). In fact, the first plot is the same as the second one, while the third plot is identical in form to the fourth one. In this instance, we actually need two scatterplots to illustrate spatial cross-correlation in empirical studies.

**Table 1:** The functional relationships of two pairs of scatterplots defined for spatial cross-correlation analysis.

Scatterplot	Abscissa (x-axis)	Ordinate (y-axis)		Effect
		Scattered points	Trend line	
The first plot	*x*	*f* ^(*y*)^ = *nWy*	*f* ^(*xy*)^ = *xy* ^T^ *Wx*	*x* acts on *y*
The second plot	*x*	*f* ^(*y*)^ = *nWy*	*f* ^(*xx*)^ = *xx* ^T^ *Wy*	*x* acts on *y*
The third plot	*y*	*f* ^(*x*)^ = *nWx*	*f* ^(*yx*)^ = *yx* ^T^ *Wy*	*y* reacts on *x*
The fourth plot	*y*	*f* ^(*x*)^ = *nWx*	*f* ^(*yy*)^ = *yy* ^T^ *Wx*	*y* reacts on *x*

The approach to making cross-correlation scatterplots is as follows. Taking *x* or *y* as an abscissa (*x*-axis) and *f* ^(*y*)^ or *f* ^(*x*)^ as an ordinate (*y*-axis), we can create a scatterplot. Then using the relationships between *x* or *y* and *f* ^(*xx*)^ or *f* ^(*xy*)^ or *f* ^(*yx*)^ or *f* ^(*yy*)^, we can produce a trendline. In short, each scatterplot includes two parts: *n* scattered points and a straight line. The relationship between *x* or *y* and *f* ^(*y*)^ or *f* ^(*x*)^ take on scattered points, but the relationship between *x* or *y* and *f* ^(*xx*)^ or *f* ^(*xy*)^ or *f* ^(*yx*)^ or *f* ^(*yy*)^ exhibit a trendline, which is in fact a regression line. In other words, the plot of *f* ^(*y*)^ or *f* ^(*x*)^ vs. *x* or *y* presents a set of randomly scattered data points, while the plot of *f* ^(*xx*)^ or *f* ^(*xy*)^ or *f* ^(*yx*)^ or *f* ^(*yy*)^ vs. *x* or *y* shows a set of ordered data points, which make a straight line. Superimposing the trendline onto the scattered data points yields a scatter diagram for spatial cross-correlation analysis.

## Discussion

### Geographical meanings of spatial cross-correlation measurements

The geographical meaning of the spatial cross-correlation can be illuminated by clarifying the mathematical relationship between Pearson’s correlation coefficient and the SCI. Leaving spatial distance out of account, we can re-express Eqs (9) and (10) as follows

$$R_{0}=x^{\text{T}}W_{0}y=y^{\text{T}}W_{0}x,$$ (33)

where *R*_0_ is the *simple correlation coefficient* (SCC), which can be treated as a special case of SCI, and

$$W_{0}=\frac{1}{n}E$$ (34)

represents a unitary identity matrix, which takes the place of the USWM, and *E* denotes an identity matrix. It can be proved that *R*_0_ is just a Pearson’s correlation coefficient:

$$R_{0}=x^{\text{T}}(\frac{1}{n}E)y=y^{\text{T}}(\frac{1}{n}E)x=\frac{1}{n}x^{\text{T}}y=\frac{1}{n}y^{\text{T}}x,$$ (35)

which indicates a simple cross-correlation between *x* and *y*. Based on Eq (35), a partial correlation coefficient can be defined as

$$R_{\text{p}}=R_{0}-R_{\text{c}}=x^{\text{T}}W_{0}y-x^{\text{T}}Wy=y^{\text{T}}W_{0}x-y^{\text{T}}Wx,$$ (36)

where *R*_p_ refers to the partial *spatial* cross-correlation coefficient (PSCC).

Now, the meanings of the spatial correlation coefficients can be explained as follows. The SCI, *R*_c_, denotes the indirect correlation between *x* and *y* through the spatial distances and other elements in a geographical system; the PSCC, *R*_p_, represents the direct cross-correlation between *x* and *y*, which is free of the spatial distance and other elements; Pearson’s correlation coefficient, *R*_0_, is a simple cross-correlation coefficient reflecting the summation of spatial correlation, including both the direct cross-correlation and the indirect cross-correlation. The SCI has two functions. First, it presents the indirect correlation between *x* and *y*, which is based on spatial distance. Second, using the indirect spatial cross-correlation coefficient, we can estimate the direct cross-correlation coefficient. Thus, the simple spatial correlation, Pearson’s correlation, can be separated into two parts: a direct correlation without distance effect and an indirect correlation based on the distance decay effect.

### Comparison between spatial autocorrelation and cross-correlation

**Fig 1:**
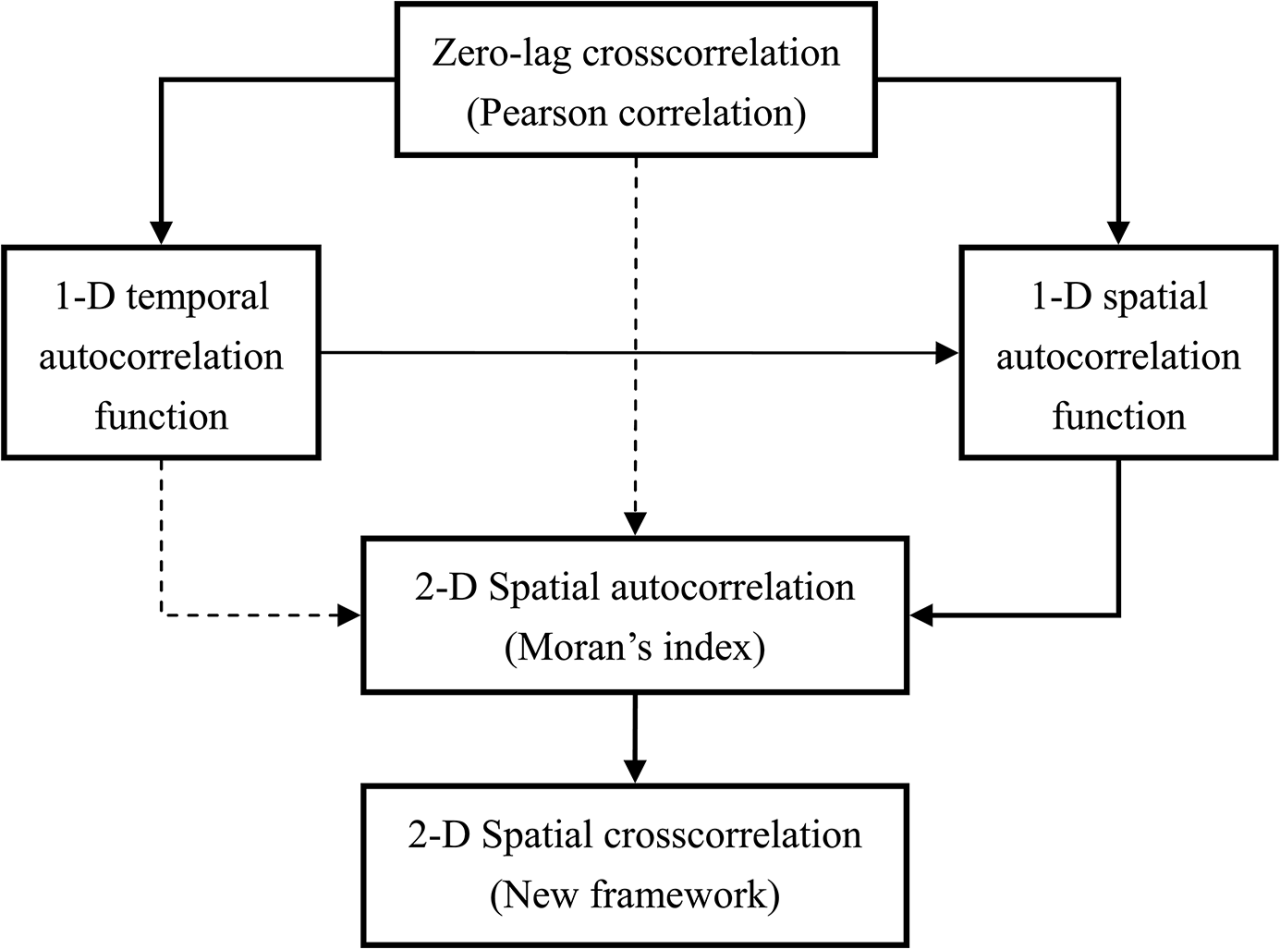
The paths from simple cross-correlation to the 2-dimensional spatial cross-correlation by way of autocorrelation (**Note**: In the block diagram, the solid line represents direct relations or paths, while the dashed line denotes the indirect relations or paths. “1-D” refers to “1-dimensional”, and “2-D” to “2-dimensional”).

For spatial analysis, autocorrelation and cross-correlation represent two different sides of the same coin. In fact, the concept of autocorrelation comes from the simplest cross-correlation, i.e. the one independent of a time lag or a spatial displacement. The autocorrelation coefficient defined in the 2-dimensional space proceeds from the autocorrelation function defined in the 1-dimensional time or space (Fig 1). The 2-dimensional cross-correlation coefficient is constructed by analogy with the 2-dimensional autocorrelation coefficient, i.e., Moran’s index, which was re-expressed in a new mathematical form [7]. A comparison can be drawn between spatial autocorrelation and spatial cross-correlation as shown in Table 2. In short, the spatial autocorrelation is the intra-sample spatial correlation, while the spatial cross-correlation is the inter-sample spatial correlation. The former is based on one size measurement, while the latter is based on two size measurements.

**Table 2:** The similarities and differences between spatial autocorrelation and spatial cross-correlation.

Item	Spatial autocorrelation	Spatial cross-correlation
Correlation property	Intra-sample correlation	Inter-sample correlation
Correlation coefficient	*I*_*x*_ = *x* ^T^ *Wx*, *I*_*y*_ = *y* ^T^ *Wy*	*R*_*xy*_ = *x* ^T^ *Wy*, *R*_*yx*_ = *y* ^T^ *Wx*
ISWM	*M* ^(*xx*)^ = *xx* ^T^ *W*, *M* ^(*yy*)^ = *yy* ^T^ *W*	*M* ^(*xy*)^ = *xy* ^T^ *W*, *M* ^(*yx*)^ = *yx* ^T^ *W*
RSWM	*M* = *nW* = *x* ^T^ *xW* = *y* ^T^ *yW*	*M* = *nW* = *x* ^T^ *xW* = *y* ^T^ *yW*
Scatterplot	One plot	Two plots

The 2-dimensional spatial correlation analyses, including spatial autocorrelation and spatial cross-correlation, are based on spatial weight matrices. A spatial weight matrix comes from a SCM, which can be generated by at least four ways [6]. For a geographical system with *n* spatial elements, a SCM can be expressed as *V* = [*v*_*ij*_], where *V* denotes the SCM, and *v*_*ij*_ is a measure used to compare and judge the degree of contiguity between place *i* and place *j* (*i*, *j* = 1,2,…,*n*). The elements on the diagonal are zeros, otherwise they must be converted into zero (i.e., for *i* = *j*, *v*_*ii*_≡0). A USWM can be defined as *w*_*ij*_ = *v*_*ij*_/*T*, where *T* denotes the sum of SCM entries. Thus, based on the *population standard deviation* (PSD), the SCI formulae, Eqs (9) and (10), can be developed in a sophisticated form as follows

$$R_{\text{c}}=\frac{X^{\text{T}}(nW)Y}{X^{\text{T}}Y}=\frac{n\sum_{i=1}^{n}\sum_{j=1}^{n}v_{ij}(X_{i}-\mu_{x})(Y_{j}-\mu_{y})}{T\sqrt{\sum_{i=1}^{n}{\left(X_{i}-\mu_{x}\right)}^{2}\sum_{i=1}^{n}{\left(Y_{i}-\mu_{y}\right)}^{2}}},$$ (37)

$$R_{\text{c}}=\frac{Y^{\text{T}}(nW)X}{Y^{\text{T}}X}=\frac{n\sum_{i=1}^{n}\sum_{j=1}^{n}v_{ij}(Y_{i}-\mu_{y})(X_{j}-\mu_{x})}{T\sqrt{\sum_{i=1}^{n}{\left(Y_{i}-\mu_{y}\right)}^{2}\sum_{i=1}^{n}{\left(X_{j}-\mu_{x}\right)}^{2}}},$$ (38)

which bear an analogy with the traditional expression of Moran’s index. If our spatial analysis is based on a sample rather than a population (universe), the PSD should be replaced by the *sample standard deviation* (SSD). In this case, the sample size *n* in Eqs (37) and (38) should be substituted by the total degree of freedom (*n*-1). For the comparability between the spatial cross-correlation index and Moran’s index, PSD rather than SSD will be employed to make empirical analyses in the next section.

## Materials and Methods

### Study area, measurements, and analytical process

The new framework of spatial cross-correlation can be employed to study the relationship between urbanization and economic development of a country. It has been confirmed that there is correlation between population urbanization and regional economic development [74]. However, the relationship between the cause and effect is not yet clear. The spatial cross-correlation analysis can be used to reveal the causality between urbanization and economic development. As an example, the spatial cross-correlation models and methods will be applied to Mainland China’s regions and cities. The spatial objects are the 31 provinces, autonomous regions, and municipalities directly under the Central Government of China and the capital cities of these regions. The level of urbanization is measured by the proportion of urban population to total population in a region, while the level of economic development is measured by the per capita gross regional product (GRP). The distances by train between any two capital cities can be used to quantify the spatial contiguity and to make a spatial weight matrix. The statistical data of urbanization levels and per capita GRP (2000–2013) are available from the website of National Bureau of Statistics (NBS) of the People's Republic of China (http://www.stats.gov.cn/tjsj/ndsj/), and the railroad distance matrix can be found in many Chinese road atlases (datasets in S1 File). Because the cities of Haikou and Lhasa were not connected to the network of Chinese cities by railway from 2000 to 2006(Lhasa)/2013(Haikou), only 29 regions and their capital cities are taken into account, and thus the size of each spatial sample is *n* = 29 (Table 3).

**Table 3:** The GRP, level of urbanization, and the LSCI values of 29 Chinese regions (2012).

*Region*	*City*	*Original variables*		*Standard variables*		*LSCI*	
		pc GRP (*X*)	Urbanization level (*Y*)	*x*	*y*	*xy* ^T^ *W* (diagonal)	*yx* ^T^ *W* (diagonal)
Beijing	Beijing	87475	86.20	2.1965	2.3931	0.0384	0.0593
Tianjin	Tianjin	93173	81.55	2.4875	2.0415	0.0589	0.0485
Hebei	Shijiazhuang	36584	46.80	-0.4029	-0.5860	-0.0061	-0.0099
Shanxi	Taiyuan	33628	51.26	-0.5539	-0.2487	-0.0021	-0.0020
Inner Mongolia	Hohehot	63886	57.74	0.9916	0.2412	0.0041	0.0009
Liaoning	Shenyang	56649	65.65	0.6220	0.8393	0.0040	0.0053
Jilin	Changchun	43415	53.70	-0.0540	-0.0642	-0.0005	-0.0003
Heilongjiang	Harbin	35711	56.90	-0.4475	0.1777	-0.0022	0.0010
Shanghai	Shanghai	85373	89.30	2.0891	2.6275	0.0099	0.0264
Jiangsu	Nanjing	68347	63.00	1.2195	0.6389	0.0134	0.0059
Zhejiang	Hangzhou	63374	63.20	0.9655	0.6541	0.0176	0.0101
Anhui	Hefei	28792	46.50	-0.8009	-0.6086	-0.0078	-0.0080
Fujian	Fuzhou	52763	59.60	0.4235	0.3819	0.0003	0.0001
Jiangxi	Nanchang	28800	47.51	-0.8005	-0.5323	-0.0018	-0.0012
Shandong	Jinan	51768	52.43	0.3727	-0.1603	0.0045	-0.0022
Henan	Zhengzhou	31499	42.43	-0.6626	-0.9164	-0.0027	-0.0042
Hubei	Wuhan	38572	53.50	-0.3013	-0.0794	0.0009	0.0002
Hunan	Changsha	33480	46.65	-0.5614	-0.5973	0.0001	0.0011
Guangdong	Guangzhou	54095	67.40	0.4915	0.9716	-0.0011	-0.0020
Guangxi	Nanning	27952	43.53	-0.8438	-0.8332	0.0013	0.0017
Chongqing	Chongqing	38914	56.98	-0.2839	0.1838	0.0025	-0.0014
Sichuan	Chengdu	29608	43.53	-0.7592	-0.8332	0.0027	0.0037
Guizhou	Guiyang	19710	36.41	-1.2648	-1.3716	0.0040	0.0066
Yunnan	Kunming	22195	39.31	-1.1378	-1.1523	0.0043	0.0047
Shaanxi	Xian	38564	50.02	-0.3017	-0.3424	0.0011	0.0010
Gansu	Lanzhou	21978	38.75	-1.1489	-1.1946	0.0058	0.0057
Qinghai	Xining	33181	47.44	-0.5767	-0.5376	0.0054	0.0046
Ningxia	Yinchuan	36394	50.67	-0.4126	-0.2933	0.0014	0.0005
Xinjiang	Urumchi	33796	43.98	-0.5453	-0.7992	0.0004	0.0006

**Note**: The original data come from National Bureau of Statistics of China (http://www.stats.gov.cn/tjsj/ndsj/). The unit of the level of urbanization is %, and the unit of GRP is *yuan* of Renminbi (RMB).

According to the theoretical model (*Results*), the analytical process of spatial cross-correlation comprises three principal steps.

**Step1**: global analysis of spatial cross-correlation. The basic measurement is the GSCI, which can be given by Eqs (9) and (10). This step is to examine the sum of spatial cross-correlation between any two regions.

**Step2**: local analysis of spatial cross-correlation. The basic measurements are the LSCIs, which can be calculated one by one using Eqs (17) and (18), or processed as batches using Eqs (19) and (20). The two vectors of LSCIs can be visually displayed with a scatterplot. This step is to investigate the spatial cross-correlation between each region and all other regions.

**Step3**: explanation of spatial cross-correlation scatterplots. Two pairs of scatterplots can be drawn using Eqs (27) to (32). Among them we need only one pair of scatterplots. Table 1 has shown the corresponding relationships between different equations. This step is to illustrate the spatial cross-correlation patterns. The local cross-correlation can be reflected by the scattered points, while the global cross-correlation can be mirrored by the trend lines.

### Calculations and analyses

The new calculation method for Moran’s index presented by Chen [7] can be adapted to the spatial cross-correlation coefficients. Based on the standardized vector *x*, *y* and the unitized weights matrix *W*, the SCI can be computed easily (an example in S2 File). The method comprises three steps as follows (an instruction in S3 File).

#### Step 1: standardize the size variables

In other words, convert the initial vectors *X*, *Y* in Eq (1) into the standardized vectors in Eq (5). As indicated above, the PSD instead of the SSD will be employed to standardize the data so that the results are comparable with Moran’s index and Pearson’s correlation coefficient. The results of 2012 are shown in Table 3.

#### Step 2: unitize the spatial weight matrix

Using the matrix of railway distances, we can compute the SCM with the distance decay function *v*(*x*) = 1/*x*, where *x* denotes the railway distance between any two capital cities. Note that the diagonal elements of the matrix should be turned into zeros. Then unitize the contiguity matrix by using the sum of the whole entries to divide each entry. The final weights matrix can be characterized with Eqs (7) and (8).

#### Step 3: compute SCI

According to Eq (9), the USWM is first left multiplied by the transpose of *x*, and then the product of *x* ^T^ and *W* is right multiplied by *y*; According to Eq (10), the unitized weights matrix is first left multiplied by the transpose of *y*, and then the product of *y* ^T^ and *W* is right multiplied by *x*. The final product of the continued multiplication yields the value of the SCI, and the two results are numerically equivalent to one another. For example, in 2012, the index of spatial cross-correlation between the level of urbanization and per capita GRP is *R*_c_ = *x* ^T^ *Wy*≈0.1566, *R*_c_ = *y* ^T^ *Wx*≈0.1566. The SCI can be separated into LSCIs, which reflect the spatial correlation between a region or city and all other regions or cities. Using Eq (19), we can calculate the first vector of the local spatial correlation coefficient, which reflects the action of *x* (economic development) on *y* (urbanization); using Eq (20), we can compute the second vector of LSCI, which reflects the reaction of *y* (urbanization) on *x* (economic development). All the results are displayed in Table 3, which shows that the sum of the LSCI equals the GSCI. The process of calculations can be fulfilled by MatLab-based computer programs (two programs in S4 File).

**Fig 2:**
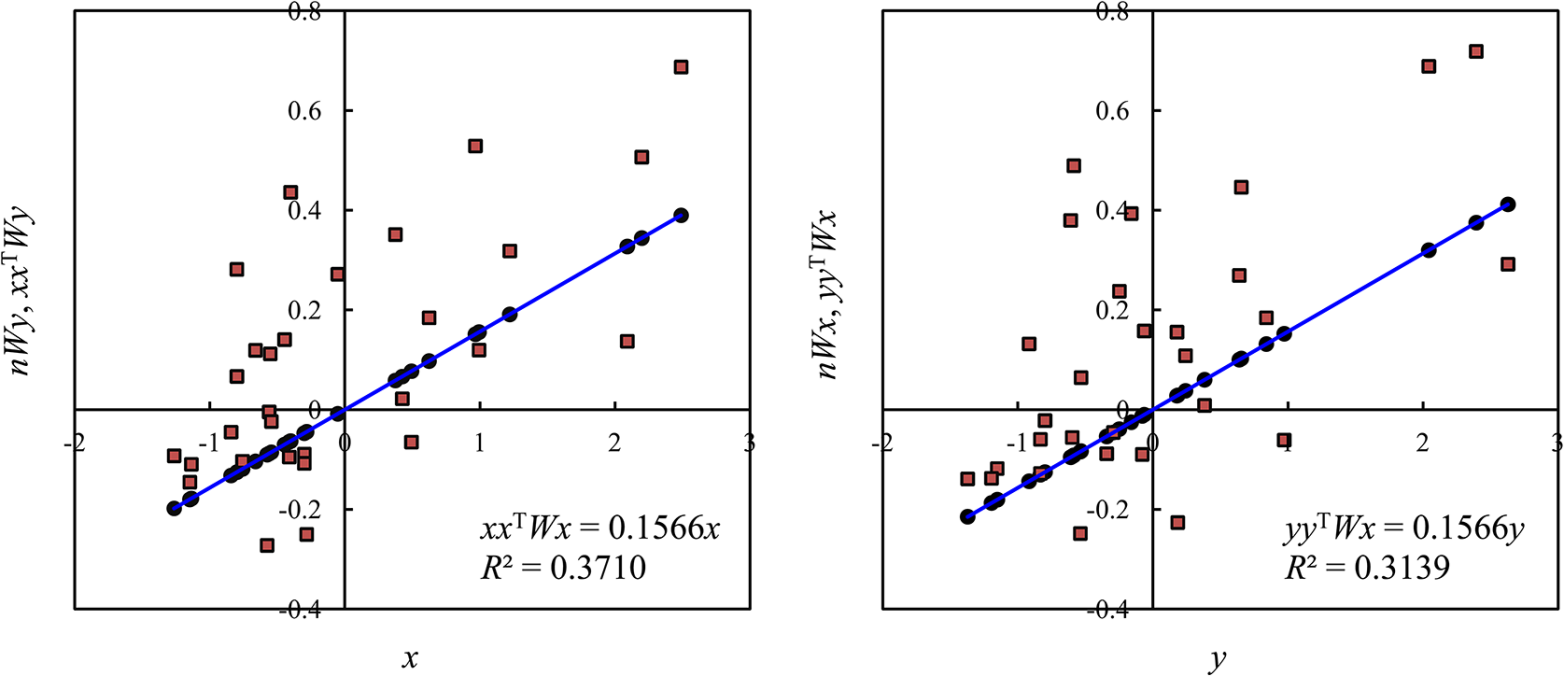
The dual scatterplots of spatial cross-correlation between the per capita GDP and the level of urbanization in cities of China (2012).

A pair of scatterplots of spatial cross-correlation can be drawn by two approaches. The first approach is to make use of the variables *x*, *y*, *nWx*, *nWy*, *xx* ^T^ *Wy*, and *yy* ^T^ *Wx*. One scatterplot is based on the relationship between *x* (*x*-axis) and *nWy* as well as *xx* ^T^ *Wy* (*y*-axis), which reflect the action of *x* (per capita GRP) on *y* (level of urbanization). The relationship between *x* and *nWy* gives the scatterpoints indicative of the first set of LSCIs, while the relationship between *x* and *xx* ^T^ *Wy* yields the trendline indicative of the GSCI (Fig 2A). The other scatterplot is based on the relationship between *y* (horizontal axis) and *nWx* as well as *yy* ^T^ *Wx* (vertical axis), which reflect the reaction of *y* (level of urbanization) on *x* (per capita GRP). The relationship between *y* and *nWx* yields the scatterpoints indicating the second set of LSCIs, while the relationship between *y* and *yy* ^T^ *Wx* gives the trendline indicating the same GSCI (Fig 2B). The second approach is to utilize the variables *x*, *y*, *nWx*, *nWy*, *xy* ^T^ *Wx*, and *yx* ^T^ *Wy*. Compared with the first approach, *xx* ^T^ *Wy* is replaced by *xy* ^T^ *Wx*, and *yy* ^T^ *Wx* is substituted with *yx* ^T^ *Wy*. The results and effects are same as those from the first approach, and the plots are the same as those displayed in Fig 2 (for 2012).

The dual scatterplots can be used to categorize Chinese cities or regions in terms of spatial cross-correlation. Each spatial cross-correlation plot includes four quadrants that indicate four basic types of geographical elements. Accordingly, Chinese regions can be grouped under four heads. The first quadrant represents the high-high (H-H) type: an element is at a higher level, and its neighbors are also at the higher level; the second quadrant represents the low-high (L-H) type: an element is at a lower level, but its neighbors are at a higher level; the third quadrant represents the low-low (L-L) type: an element is at a lower level, and its neighbors are also at the lower level; the fourth quadrant represents the high-low (H-L) type: an element is at a high level, but its neighbors are at a lower level. Where 2012 year is concerned, the classification result based on the first scatterplot is generally consistent with that based on the second scatterplot (Table 4). Only three regions are uncertain, that is, Chongqing (municipality), Heilongjiang (province), and Shandong (province). This suggests that the three regions are at the edges of different types of economic and urban zones.

**Table 4:** The classification results of China’s regions based on the dual spatial cross-correlation scatterplots of urbanization and economic development (2012).

*Quadrant*	*Type*	*Economic development acts on urbanization (x vs. nWy,)*		*Urbanization reacts on economic development (y vs. nWx)*	
		Regions	Number	Regions	Number
First one	H-H	Beijing, Fujian, Inner Mongolia, Jiangsu, Liaoning, *Shandong*, Shanghai, Tianjin, Zhejiang	9	Beijing, Fujian, *Heilongjiang*, Inner Mongolia, Jiangsu, Liaoning, Shanghai, Tianjin, Zhejiang	9
Second one	L-H	Anhui, Hebei, *Heilongjiang*, Henan, Jilin, Jiangxi, Shanxi	7	Anhui, Hebei, Henan, Jilin, Jiangxi, *Shandong*, Shanxi	7
Third one	L-L	*Chongqing*, Gansu, Guangxi, Guizhou, Hubei, Hunan, Ningxia, Qinghai, Shaanxi, Sichuan, Xinjiang, Yunnan	12	Gansu, Guangxi, Guizhou, Hubei, Hunan, Ningxia, Qinghai, Shaanxi, Sichuan, Xinjiang, Yunnan	11
Fourth one	H-L	Guangdong	1	*Chongqing*, Guangdong	2

**Note**: The regions that are expressed in italic type represent the uncertain elements.

**Fig 3:**
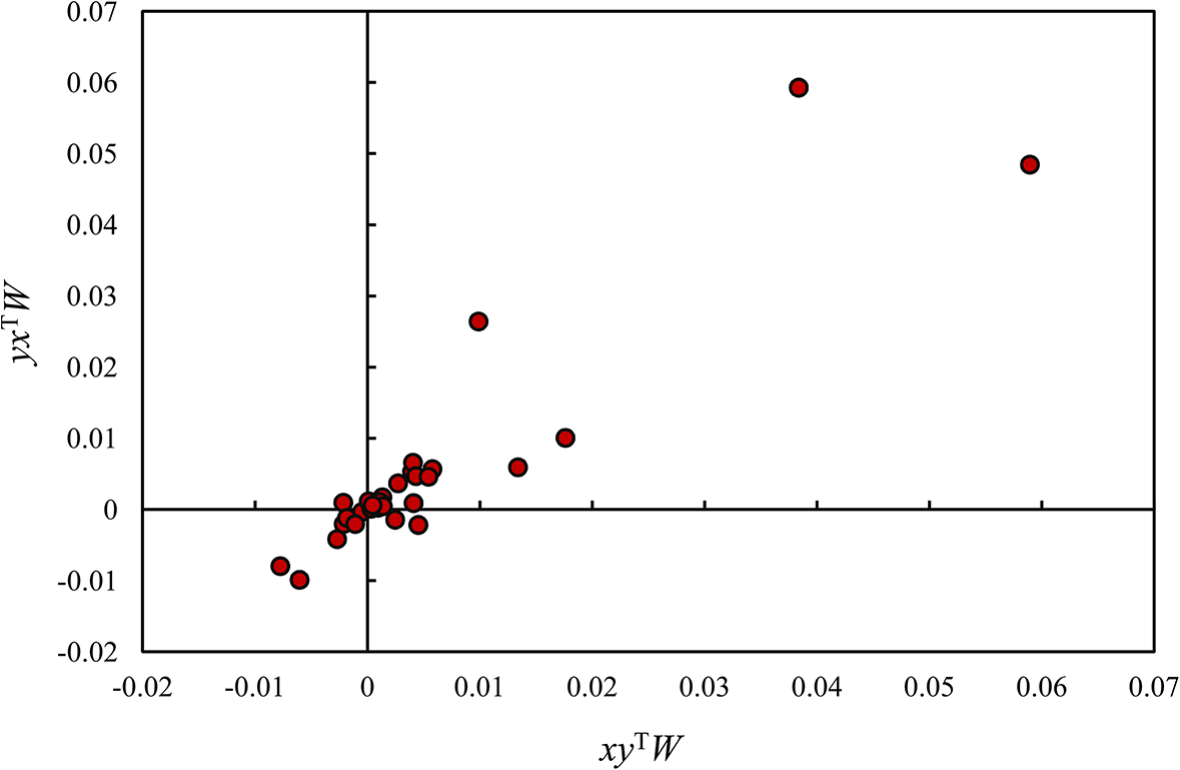
The scatterplot of local spatial cross-correlation between the per capita GRP and the level of urbanization in regions of China (2012).

The relationship between the two sets of LSCIs can also be shown by a scatterplot. The plot is a visual aid for categorizing Chinese regions (Fig 3). As far as 2012 year is concerned, the 29 Chinese regions can be distributed into 4 groups according to the quadrants of a Cartesian coordinate system. This classification process rearranges the results given by the dual cross-correlation scatterplots. The H-H type and L-L type such as Beijing, Tianjin, and Shanghai are in the first quadrant, the L-H type such as Anhui, Hebei and the H-L type such as Guangdong are in the third quadrant, and the uncertain type including Chongqing, Heilongjiang, and Shandong are in the second and fourth quadrants (Table 5). Apparently, the LSCI scatterplot lends further support to the clustering result from the spatial cross-correlation scatterplots.

**Table 5:** The classification results of China’s regions based on local cross-correlation indexes of urbanization and economic development (2012).

Quadrant	Region	Type
First	Beijing, Fujian, Gansu, Guangxi, Hubei, Hunan, Jiangsu, Ningxia, Qinghai, Shaaxi, Shanghai, Tianjin, Xinjiang, Zhejiang	H-H, L-L
Second	Heilongjiang	Uncertain
Third	Anhui, Guangdong, Hebei, Henan, Jiangxi, Jilin, Shanxi	L-H, H-L
Fourth	Chongqing, Shandong	Uncertain

A difficult problem about the relationship between urbanization and economic development is to reveal the causality. This problem can be solved by spatial cross-correlation analysis. In the cross-correlation scatterplots, the slopes of the trend lines equal the SCI value. This suggests that we can employ the regression analysis based on the least squares method to estimate the SCI using Eqs (21) and (22). A discovery is that Eq (21) and Eq (22) give the same SCI value (*R*_c_), but the values of goodness of fit (*R* ^2^) are different. If the independent variable is *x*, the dependent variable will be *nWy*. For 2012, the SCI value is about *R*_c_ = 0.1566, and the coefficient of determination is approximately *R* ^2^ = 0.3710. The standard error of is about *δ* = 0.0385. This suggests that the per capita GRP can explain about 37.10% of the spatial change of the level of urbanization. If the independent variable is *y*, the dependent variable will be *nWx*. For 2012, the SCI value is still about *R*_c_ = 0.1566, but the determination coefficient is approximately *R* ^2^ = 0.3139. The standard error of is around *δ* = 0.0438. This suggests that the level of urbanization can explain about 31.39% of the spatial change of the per capita GRP. Note that the intercept should be set to 0 as there is no constant term in the regression equations abovementioned.

Different coefficients of determination result in different values of *F* statistic, *t* statistics, and standard errors. The *F* statistics can be used to judge the cause and effect, the *t* statistics can be utilized to judge the level of confidence of a model’s parameter, and the standard errors can be employed to estimate the margin of error of a coefficient. For the regression analysis with a single explanatory variable, the *F* statistic, *t* statistics, and parameter standard errors (*δ*) are all equivalent to the *R* square, and can be computed by the following formulae\

$$F=t^{2}=\frac{vR^{\text{2}}}{1-R^{2}}\;,$$ (39)

$$\delta =R_{c}\sqrt{\frac{1}{v}(\frac{1}{R^{2}}-1)}\;,$$ (40)

where *v* refers to the residual degree of freedom. For our example, because the intercept (constant item) is zero, the degree of freedom is actually *v* = *n*-1 = 28. Given *R* ^2^ = 0.3710, it follows that *F* = 16.5136, *t* = 4.0637, *δ* = 0.0385; If *R* ^2^ = 0.3139 as given, then *F* = 12.8075, *t* = 3.5788, *δ* = 0.0438. Accordingly, the significance for *δ* = 0.0385 is about *p* = 0.0004, and that for *δ* = 0.0438 is about *p* = 0.0013 (Table 6) (see the example in S2 File).The rest may be deduced by analogy with these. In a linear regression analysis, the *F* statistic indicates the extent to which an independent variable can explain the corresponding dependent variable. For 2012 year, the action of *x* on *y* (*R* ^2^ = 0.3710, *F* = 16.5136) is stronger than the reaction of *y* on *x* (*R* ^2^ = 0.3139, *F* = 12.8075). This seems to suggest that the influence of economic development on urbanization is greater than the impact of urbanization on economic development.

**Table 6:** The values of SCC, SCI, PSCC and determination coefficients of the 29 Chinese regions (2000–2013).

Year	2000	2005	2006	2007	2008	2009	2010	2011	2012	2013
SCC *R*_0_	0.9142	0.9451	0.9447	0.9470	0.9523	0.9512	0.9577	0.9520	0.9457	0.9428
SCI *R*_c_	0.0995	0.1382	0.1409	0.1415	0.1521	0.1550	0.1566	0.1575	0.1566	0.1556
PSCC *R*_p_	0.8147	0.8068	0.8038	0.8056	0.8001	0.7962	0.8011	0.7945	0.7891	0.7872
*R* ^2^_(*x*-*y*)_	0.1704	0.2940	0.3031	0.3015	0.3431	0.3499	0.3750	0.3756	0.3710	0.3623
*R* ^2^_(*y*-*x*)_	0.1540	0.2450	0.2544	0.2576	0.2846	0.2975	0.3056	0.3133	0.3139	0.3189
*R* ^2^_(*x*-*y*)_+ *R* ^2^_(*y*-*x*)_	0.3244	0.5391	0.5575	0.5591	0.6277	0.6474	0.6807	0.6889	0.6848	0.6813
*F*_(*x*-*y*)_	5.7531	11.6628	12.1773	12.0834	14.6235	15.0728	16.8033	16.8437	16.5136	15.9081
*δ*_(*x*-*y*)_	0.0415	0.0405	0.0404	0.0407	0.0398	0.0399	0.0382	0.0384	0.0385	0.0390
*P*_(*x*-*y*)_	0.0234	0.0020	0.0016	0.0017	0.0007	0.0006	0.0003	0.0003	0.0004	0.0004
*F*_(*y*-*x*)_	5.0965	9.0879	9.5518	9.7173	11.1373	11.8579	12.3233	12.7729	12.8075	13.1127
*δ*_(*y*-*x*)_	0.0441	0.0459	0.0456	0.0454	0.0456	0.0450	0.0446	0.0441	0.0438	0.0430
*P*_(*y*-*x*)_	0.0320	0.0054	0.0045	0.0042	0.0024	0.0018	0.0015	0.0013	0.0013	0.0011

**Note**: The statistical data of the level of urbanization from 2001 to 2004 are absent in the website of China’s NBS. The statistic *R* ^2^_(*x*-*y*)_ denotes the goodness of fit for the regression of *nWy* depending on *x*, and *R* ^2^_(*y*-*x*)_ refers to the goodness of fit for the regression of *nWx* depending on *y*. Based on the *R* ^2^ values, the *F* statistics, *t* statistics, and parameter standard errors *δ* can be calculated with the formulae such as *F* = *t* ^2^ = (*n*-1)*R* ^2^/(1-*R* ^2^) and *δ* = *R*_c_[(1/*R* ^2^-1)/(*n*-1)]^1/2^. Further, the significance, *P*, can be reckoned using the *F* distribution function of MS Excel.

The coefficient of simple correlation between the level of urbanization and that of economic development of Mainland China can be decomposed by using the SCI value, and thus we obtain direct correlation coefficients. For example, for 2012, the Pearson correlation coefficient can be calculated with Eq (35), and the result is about *R*_0_ = 0.9457. Then, according to Eq (36), the PSCC is about *R*_p_ = 0.9457–0.1566 = 0.7891. A conclusion can be drawn from these values of correlation coefficients that the direct correlation index of the 29 regions is 0.7891 or so, and the indirect correlation index is round about 0.1566. The former has little relation to the distances between different provincial capital cities and can be regarded as intra-group correlation, but the latter is related to spatial interaction of different regions based on distances and can be treated as intergroup correlation.

Similarly, the analytical process of spatial cross-correlation can be further applied one by one to the other datasets of the years from 2000 to 2013. The parameters include SCC (*R*_0_), SCI (*R*_c_), and PSCC (*R*_p_), and the goodness of fit for the regression analyses of spatial cross-correlation have been computed (Table 6). The related statistics can be evaluated with Eqs (39) and (40). From these calculations, we can get useful spatio-temporal information for China’s urbanization and economic development.

#### First, there is weak positive spatial cross-correlation between Chinese per capita GRP and the level of urbanization

The SCI values come between 0.0995 and 0.1575. The Pearson correlation coefficients (SCC) range from 0.9142 to 0.9577. Thus the partial correlation coefficients (PSCC) vary from 0.7872 to 0.8147. This suggests that the correlation between urbanization and economic development includes an influence factor from spatial interaction.

#### Second, the spatial cross-correlation between urbanization and economic development of China became stronger and stronger

**Fig 4:**
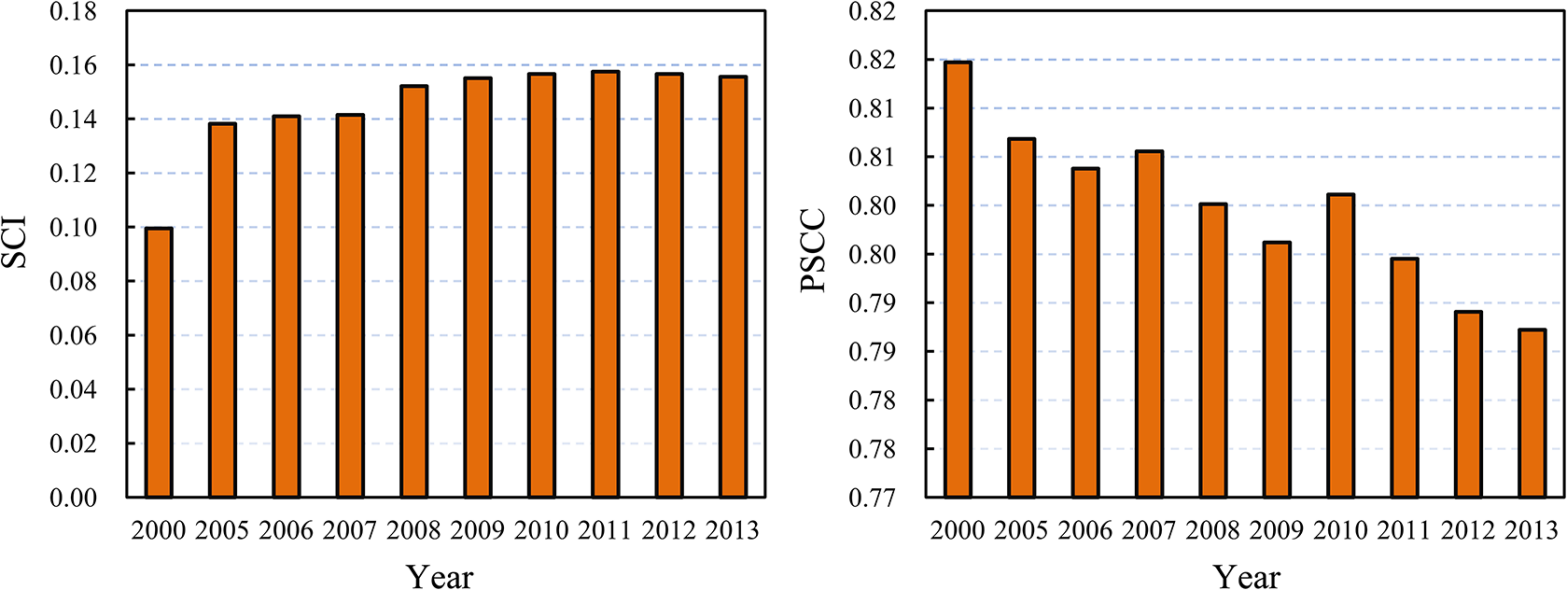
Histograms of SCI and PSCC of the spatial cross-correlation of 29 Chinese regions (2000–2013).

The simple correlation is relatively stable, and the SCC values fluctuate around 0.95. However, the SCI values went up and up, while the PSCC values went down gradually (Fig 4). This suggests that the spatial interaction between different regions and cities became more and more significant in the process of spatio-temporal evolution of China’s regional systems.

#### Third, the action of economic development on urbanization is relatively stronger than the reaction of urbanization on economic development

**Fig 5:**
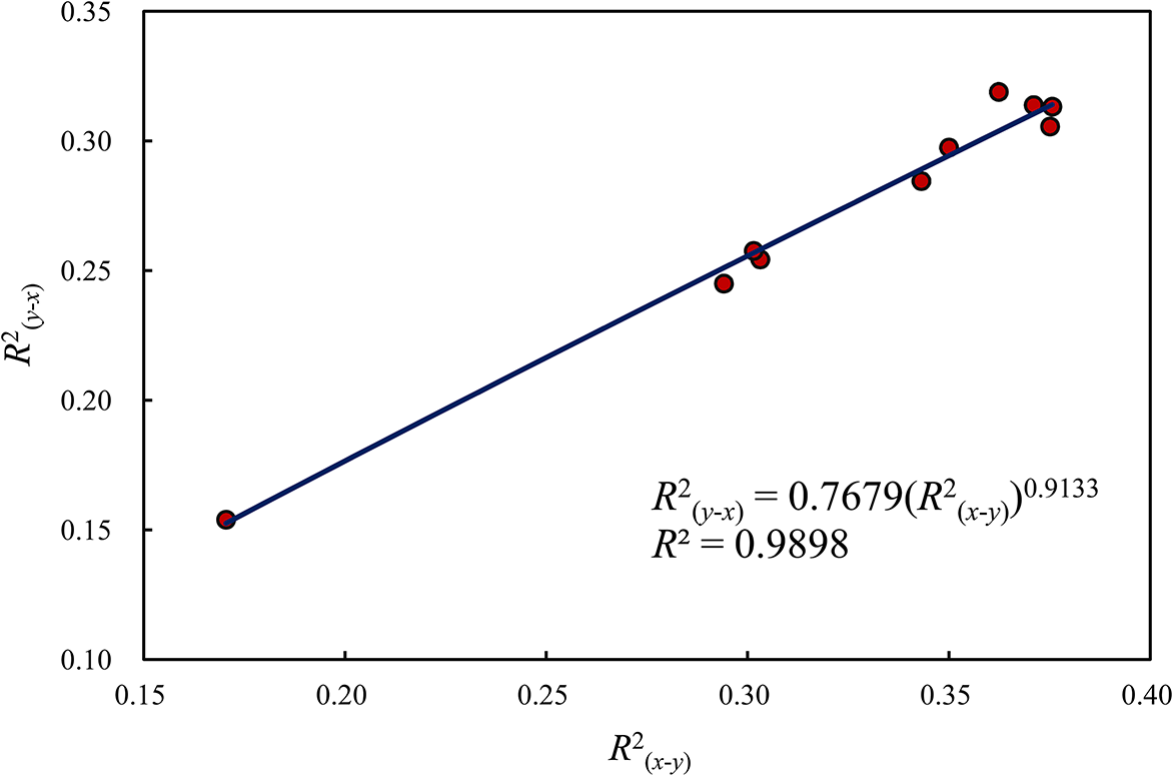
The allometric relationship between two kinds of determination coefficients for spatial cross-correlation analysis (2000–2013).

The goodness of fit for the regression of *nWy* depending on *x*, *R* ^2^_(*y*-*x*)_, is all greater than that for the regression of *nWx* depending on *y*, *R* ^2^_(*x*-*y*)_. This suggests that economic development is a cause of urbanization, and urbanization is an effect of economic development. On the whole, both the values of *R* ^2^_(*x*-*y*)_ and *R* ^2^_(*y*-*x*)_ go up and up from 2000 to 2013. This lends support to the inference that the spatial interaction of the 29 regions became more and more significant over time. The absolute and relative growth rates of *R* ^2^_(*y*-*x*)_ are less than those of *R* ^2^_(*x*-*y*)_. The relationship between the relative growth rate of *R* ^2^_(*y*-*x*)_ and that of *R* ^2^_(*x*-*y*)_ can be shown by the allometric scaling pattern (Fig 5). The allometric exponent of *R* ^2^_(*y*-*x*)_ depending on *R* ^2^_(*x*-*y*)_ is about 0.9133, which is less than 1. The *R* ^2^ values can be converted into *F* statistics using the hyperbolic function, Eq (39), and we have *F*_(*x*-*y*)_>*F*_(*y*-*x*)_. The *F* statistics imply that the explanation of economic development for urbanization is more than that of urbanization for economic development. This lends further support to the proposition that the level of urbanization in a geographical region is determined by the level of economic development and in turn reacts to it.

## Conclusions

This paper is devoted to laying the foundation for development of spatial cross-correlation theory. The basic measurements and analytical methods are put forward and applied to an urban study of China. In terms of technology, two computer programs based on MatLab have been written and provided for readers. On the basis of the theoretical results and empirical analyses, three basic conclusions can be drawn as follows.

### First, spatial autocorrelation and spatial cross-correlation can complement one another

Both autocorrelation and cross-correlation analyses can be employed to study the correlation effect of different spatial elements in a regional system or different subsystems within a geographical system. The two methods are different, but they can combine to make an integrated framework. The spatial autocorrelation analysis shows the simultaneous change in value of one random variable, while the spatial cross-correlation analysis displays the simultaneous change in values of two random variables. If we use one variable to measure a number of spatial entities, we can make a spatial autocorrelation analysis; on the other hand, if we use two or more variables to measure a number of spatial entities, we can make both spatial autocorrelation analysis and spatial cross-correlation analysis.

### Second, the spatial cross-correlation coefficient represents the indirect relationships between spatial variables

Using SCI, we can analyze the well-known simple correlation coefficient in spatial statistics. Pearson’s correlation between two spatial variables includes two components: direct correlation and indirect correlation. The spatial correlation coefficient reflects the indirect correlation based on the spatial contiguity between any two geographical entities. Pearson’s correlation coefficient minus the spatial cross-correlation coefficient leaves the direct correlation coefficient. The direct correlation is actually a kind of partial correlation, which is independent of spatial patterns. In this sense, spatial cross-correlation analysis can reveal the importance of the part played by geographical distances or spatial relationships.

### Third, the dual scatterplots of spatial cross-correlation can be used to reveal the causality between two variables visually

Pearson’s correlation coefficient and spatial cross-correlation coefficient can reflect the correlation between two variables, but they cannot distinguish between cause and effect. The scatterplots of spatial cross-correlation can be used to differentiate between the cause and the effect. The spatial cross-correlation plots appear by twos, and the two plots are of asymmetry. Therefore, they can show us which variable is in the leading position and which is in the subordinate position. In scientific research, determining causality may be more important than describing correlation in a system. Moreover, the scatterplots can serve for an assistant approach to making a spatial classification of geographical elements.

## Supporting Information

S1 File Datasets of per capita GRP, level of urbanization, and railway distances. This file contains the original or preliminarily processed data used in this paper.
(XLSX)

S2 File The calculation process of SCI for 2012 (example). It provides two complete processes of computing the spatial cross-correlation coefficients based on power-law decay and exponential decay, respectively.
(XLSX)

S3 File An instruction for calculating SCI using MS Excel. It illustrates how to calculate a spatial cross-correlation coefficient step by step using MS Excel.
(PDF)

S4 File Two matlab programs for spatial cross-correlation analysis. It provides two MatLab programs for calculating spatial cross-correlation coefficients: one is based on the power-law decay function, and the other is based on the exponential-decay function. Readers can employ the programs to carry out spatial cross-correlation analyses by substituting the data with their own data.
(M) Click here for additional data file.
