# Supplementary material for: A New Methodology of Spatial Cross-Correlation Analysis
Source: PLoS One. 2015 May 19;10(5):e0126158. doi: 10.1371/journal.pone.0126158 (PMC4438072; doi:10.1371/journal.pone.0126158)
Supplement: S3 File — It illustrates how to calculate a spatial cross-correlation coefficient step by step using MS Excel. (PDF) [file pone.0126158.s003.pdf]

An instruction for calculating SCI using MS Excel

The spatial crosscorrelation index (SCI) can be calculated with MS Excel. The basic steps are similar to those that are employed to compute Moran's index (Chen, 2013). Taking 29 Chinese regions, including provinces, autonomous regions, and municipalities directly under the central government, as an example, I will show how to evaluate the SCI using what is called *three-step method*.

1 Preparation of data

First of all, two datasets must be prepared as below: one is the vectors of per capita *gross regional product* (GRP) and *level of urbanization* (Figure S1), and the other is the railway distance matrix of Chinese capital cities (Figure S2).

	A	B	C
1	**Region**	**GRP(*yuan*)**	**Level of urbanization(%)**
2	Beijing	87475	86.20
3	Tianjin	93173	81.55
4	Hebei	36584	46.80
5	Shanxi	33628	51.26
6	Inner Mongolia	63886	57.74
7	Liaoning	56649	65.65
8	Jilin	43415	53.70
9	Heilongjiang	35711	56.90
10	Shanghai	85373	89.30
11	Jiangsu	68347	63.00
12	Zhejiang	63374	63.20
13	Anhui	28792	46.50
14	Fujian	52763	59.60
15	Jiangxi	28800	47.51
16	Shandong	51768	52.43
17	Henan	31499	42.43
18	Hubei	38572	53.50
19	Hunan	33480	46.65
20	Guangdong	54095	67.40
21	Guangxi	27952	43.53
22	Chongqing	38914	56.98
23	Sichuan	29608	43.53
24	Guizhou	19710	36.41
25	Yunnan	22195	39.31
26	Shaanxi	38564	50.02
27	Gansu	21978	38.75
28	Qinghai	33181	47.44
29	Ningxia	36394	50.67
30	Xinjiang	33796	43.98

Figure S1 The statistical data of GRP and urbanization level of 29 Chinese regions in 2012

	G	H	I	J	K	L	M	N	O	P	Q
1		Beijing	Tianjin	Shijiazhuang	Taiyuan	Hohehot	Shenyang	Changchun	Harbin	Shanghai	Nanjing
2	Beijing	0	137	277	508	667	741	1046	1288	1463	1160
3	Tianjin	137	0	419	650	804	728	1033	1275	1326	1023
4	Shijiazhuang	277	419	0	231	871	1126	1431	1673	1267	964
5	Taiyuan	508	650	231	0	640	1255	1560	1802	1498	1195
6	Hohehot	667	804	871	640	0	1408	1713	1955	2130	1827
7	Shenyang	741	728	1126	1255	1408	0	305	547	2054	1751
8	Changchun	1046	1033	1431	1560	1713	305	0	242	2359	2056
9	Harbin	1288	1275	1673	1802	1955	547	242	0	2601	2298
10	Shanghai	1463	1326	1267	1498	2130	2054	2359	2601	0	303
11	Nanjing	1160	1023	964	1195	1827	1751	2056	2298	303	0
12	Hangzhou	1589	1452	1393	1624	2256	2180	2485	2727	201	429
13	Hefei	1074	973	914	1145	1777	1701	2006	2248	615	312
14	Fuzhou	2334	2197	1915	2521	3303	2925	3230	3472	1180	1174
15	Nanchang	1449	1444	1293	1544	2674	2151	2456	2698	844	838
16	Jinan	497	360	301	532	1164	1088	1393	1635	966	663
17	Zhengzhou	689	831	412	577	1362	1538	1843	2085	998	695
18	Wuhan	1225	1367	948	1179	1898	1972	2277	2519	1235	1231
19	Changsha	1587	1729	1310	1537	2256	2334	2639	2881	1207	1201
20	Guangzhou	2294	2436	2017	2243	2962	3041	3346	3588	1810	1804
21	Nanning	2566	2708	2289	2515	3234	3313	3618	3860	2082	2076
22	Chongqing	2087	2230	1810	1997	2637	2834	3139	3381	2516	2093
23	Chengdu	2042	2185	1765	1493	2133	2789	3094	3336	2351	2048
24	Guiyang	2539	2681	2262	2460	3100	3286	3591	3833	2053	2054
25	Kunming	3178	3320	2901	2593	3233	3925	4230	4472	2699	2693
26	Xian	1159	1301	923	651	1291	1906	2211	2453	1509	1206
27	Lanzhou	1811	1948	1599	1327	1144	2552	2857	3099	2185	1882
28	Xining	2092	2235	1815	1543	1360	2839	3144	3386	2401	2098
29	Yinchuan	1343	1480	1547	1316	676	2084	2389	2631	2355	2052
30	Urumchi	3768	3911	3491	3219	3036	4515	4820	5062	4077	3774

Figure S2 The railroad distance matrix of 29 Chinese cities by railway network (partial results)

2 Process of calculations based on power function

2.1 Step 1: standardizing size measures

The procedure of variable standardization comprises three steps. **First**, compute the mean value of the GRP and the level of urbanization using the MS Excel function “average”. In cell B31, input a formula in the form “=AVERAGE(B2:B30)”, press the **Enter** key, and it will yield the GRP mean about 44471.586; In cell C31, input a formula “=AVERAGE(C2:C30)” and press **Enter**, and it will give the mean of urbanization level about 54.550. **Second**, compute the *population standard deviation* (PSD) using the Excel function “stdevp”. In cell B32, input a formula “=STDEVP(B2:B30)”, press **Enter**, yield a PSD value about 19578.182; In cell C32, input a formula “=STDEVP(C2:C30)”, press **Enter**, generate a PSD value around 13.226. **Third**, standardize the population size. Select a region in the worksheet including cells D2-D30, input a formula “=STANDARDIZE(B2:B30,B31,B32)”, press **Ctrl** and **Shift** and **Enter** at the same time,

thus yield the standardized array indicative of the GRP vector (*x*); Select another region including cells E2-E30, input a formula "=STANDARDIZE(C2:C30,C31,C32)", press **Ctrl** and **Shift** and then **Enter**, it generates the standardized array indicating the vector of urbanization level (*y*). The results are displayed in Figure S3 (see File S2 for details).

	A	B	C	D	E
1	**Region**	**GRP(*yuan*)**	**Level of urbanization(%)**	***x***	***y***
2	Beijing	87475	86.20	2.1965	2.3931
3	Tianjin	93173	81.55	2.4875	2.0415
4	Hebei	36584	46.80	-0.4029	-0.5860
5	Shanxi	33628	51.26	-0.5539	-0.2487
6	Inner Mongolia	63886	57.74	0.9916	0.2412
7	Liaoning	56649	65.65	0.6220	0.8393
8	Jilin	43415	53.70	-0.0540	-0.0642
9	Heilongjiang	35711	56.90	-0.4475	0.1777
10	Shanghai	85373	89.30	2.0891	2.6275
11	Jiangsu	68347	63.00	1.2195	0.6389
12	Zhejiang	63374	63.20	0.9655	0.6541
13	Anhui	28792	46.50	-0.8009	-0.6086
14	Fujian	52763	59.60	0.4235	0.3819
15	Jiangxi	28800	47.51	-0.8005	-0.5323
16	Shandong	51768	52.43	0.3727	-0.1603
17	Henan	31499	42.43	-0.6626	-0.9164
18	Hubei	38572	53.50	-0.3013	-0.0794
19	Hunan	33480	46.65	-0.5614	-0.5973
20	Guangdong	54095	67.40	0.4915	0.9716
21	Guangxi	27952	43.53	-0.8438	-0.8332
22	Chongqing	38914	56.98	-0.2839	0.1838
23	Sichuan	29608	43.53	-0.7592	-0.8332
24	Guizhou	19710	36.41	-1.2648	-1.3716
25	Yunnan	22195	39.31	-1.1378	-1.1523
26	Shaanxi	38564	50.02	-0.3017	-0.3424
27	Gansu	21978	38.75	-1.1489	-1.1946
28	Qinghai	33181	47.44	-0.5767	-0.5376
29	Ningxia	36394	50.67	-0.4126	-0.2933
30	Xinjiang	33796	43.98	-0.5453	-0.7992
31	**Average**	**44471.586**	**54.550**	**0.000**	**0.000**
32	**Stdevp**	**19578.182**	**13.226**	**1.000**	**1.000**

Figure S3 The standardized variables of GRP and urbanization levels of 29 Chinese regions

2.2 Step 2: generating unitary spatial weights matrix

The process of generating a spatial weights matrix also consists of three steps. **First**, convert the spatial distance matrix into a spatial contiguity matrix. Select an impedance function based on the inverse power law such as

$$v_{ij} = \begin{cases} r_{ij}^{-1}, & i \neq j \\ 0, & i = j \end{cases}, \qquad (1)$$

where r_{ij} denotes the distance between city i and city j, and v_{ij} represents the spatial contiguity measurement. In cell H33, input a formula such as "=IF(F2=0,0,1/H2)", press **Enter**, it will yield a number 0. Seizing the lower right corner of cell H33 with the mouse, and dragging it right and down, we can generate all the spatial contiguity values, which are partially shown in Figure S4. **Second**, summate the spatial contiguity values using the following formula:

$$T = \sum_i \sum_j v_{ij} , \tag{2}$$

where T denotes an amount obtained by double summation. In cell AJ62, input a formula "=SUM(H33:AJ61)", strike **Enter** key, yield a sum about 0.6296. **Third**, transform the spatial contiguity matrix into a *unitized spatial weights matrix* (W). In cell H64, input a formula "=H33/$AJ$62", press **Enter**, and it will yield the first value of spatial weights. Catching the bottom right corner of cell H64, pulling it right and down, we can produce all the values of the unitary spatial weights matrix, which are displayed in Figure S5 (see File S2 for details).

	G	H	I	J	K	L	M	N	O	P	Q
32		Beijing	Tianjin	Shijiazhuang	Taiyuan	Hohehot	Shenyang	Changchun	Harbin	Shanghai	Nanjing
33	Beijing	0	0.0072993	0.0036101	0.0019685	0.0014993	0.0013495	0.000956	0.0007764	0.0006835	0.0008621
34	Tianjin	0.0072993	0	0.0023866	0.0015385	0.0012438	0.0013736	0.0009681	0.0007843	0.0007541	0.0009775
35	Shijiazhuang	0.0036101	0.0023866	0	0.004329	0.0011481	0.0008881	0.0006988	0.0005977	0.0007893	0.0010373
36	Taiyuan	0.0019685	0.0015385	0.004329	0	0.0015625	0.0007968	0.000641	0.0005549	0.0006676	0.0008368
37	Hohehot	0.0014993	0.0012438	0.0011481	0.0015625	0	0.0007102	0.0005838	0.0005115	0.0004695	0.0005473
38	Shenyang	0.0013495	0.0013736	0.0008881	0.0007968	0.0007102	0	0.0032787	0.0018282	0.0004869	0.0005711
39	Changchun	0.000956	0.0009681	0.0006988	0.000641	0.0005838	0.0032787	0	0.0041322	0.0004239	0.0004864
40	Harbin	0.0007764	0.0007843	0.0005977	0.0005549	0.0005115	0.0018282	0.0041322	0	0.0003845	0.0004352
41	Shanghai	0.0006835	0.0007541	0.0007893	0.0006676	0.0004695	0.0004869	0.0004239	0.0003845	0	0.0033003
42	Nanjing	0.0008621	0.0009775	0.0010373	0.0008368	0.0005473	0.0005711	0.0004864	0.0004352	0.0033003	0
43	Hangzhou	0.0006293	0.0006887	0.0007179	0.0006158	0.0004433	0.0004587	0.0004024	0.0003667	0.0049751	0.002331
44	Hefei	0.0009311	0.0010277	0.0010941	0.0008734	0.0005627	0.0005879	0.0004985	0.0004448	0.001626	0.0032051
45	Fuzhou	0.0004284	0.0004552	0.0005222	0.0003967	0.0003028	0.0003419	0.0003096	0.000288	0.0008475	0.0008518
46	Nanchang	0.0006901	0.0006925	0.0007734	0.0006477	0.000374	0.0004649	0.0004072	0.0003706	0.0011848	0.0011933
47	Jinan	0.0020121	0.0027778	0.0033223	0.0018797	0.0008591	0.0009191	0.0007179	0.0006116	0.0010352	0.0015083
48	Zhengzhou	0.0014514	0.0012034	0.0024272	0.0017331	0.0007342	0.0006502	0.0005426	0.0004796	0.001002	0.0014388
49	Wuhan	0.0008163	0.0007315	0.0010549	0.0008482	0.0005269	0.0005071	0.0004392	0.000397	0.0008097	0.0008123
50	Changsha	0.0006301	0.0005784	0.0007634	0.0006506	0.0004433	0.0004284	0.0003789	0.0003471	0.0008285	0.0008326
51	Guangzhou	0.0004359	0.0004105	0.0004958	0.0004458	0.0003376	0.0003288	0.0002989	0.0002787	0.0005525	0.0005543
52	Nanning	0.0003897	0.0003693	0.0004369	0.0003976	0.0003092	0.0003018	0.0002764	0.0002591	0.0004803	0.0004817
53	Chongqing	0.0004792	0.0004484	0.0005525	0.0005008	0.0003792	0.0003529	0.0003186	0.0002958	0.0003975	0.0004778
54	Chengdu	0.0004897	0.0004577	0.0005666	0.0006698	0.0004688	0.0003586	0.0003232	0.0002998	0.0004254	0.0004883
55	Guiyang	0.0003939	0.000373	0.0004421	0.0004065	0.0003226	0.0003043	0.0002785	0.0002609	0.0004871	0.0004869
56	Kunming	0.0003147	0.0003012	0.0003447	0.0003857	0.0003093	0.0002548	0.0002364	0.0002236	0.0003705	0.0003713
57	Xian	0.0008628	0.0007686	0.0010834	0.0015361	0.0007746	0.0005247	0.0004523	0.0004077	0.0006627	0.0008292
58	Lanzhou	0.0005522	0.0005133	0.0006254	0.0007536	0.0008741	0.0003918	0.00035	0.0003227	0.0004577	0.0005313
59	Xining	0.000478	0.0004474	0.000551	0.0006481	0.0007353	0.0003522	0.0003181	0.0002953	0.0004165	0.0004766
60	Yinchuan	0.0007446	0.0006757	0.0006464	0.0007599	0.0014793	0.0004798	0.0004186	0.0003801	0.0004246	0.0004873
61	Urumchi	0.0002654	0.0002557	0.0002865	0.0003107	0.0003294	0.0002215	0.0002075	0.0001976	0.0002453	0.000265

Figure S4 The spatial contiguity matrix of 29 Chinese capital cities based on power function (partial results)

	G	H	I	J	K	L	M	N	O	P	Q
63		Beijing	Tianjin	Shijiazhuang	Taiyuan	Hohehot	Shenyang	Changchun	Harbin	Shanghai	Nanjing
64	Beijing	0	0.0115931	0.0057338	0.0031265	0.0023812	0.0021434	0.0015184	0.0012331	0.0010856	0.0013692
65	Tianjin	0.0115931	0	0.0037906	0.0024435	0.0019754	0.0021817	0.0015375	0.0012457	0.0011978	0.0015525
66	Shijiazhuang	0.0057338	0.0037906	0	0.0068755	0.0018235	0.0014105	0.0011099	0.0009493	0.0012536	0.0016476
67	Taiyuan	0.0031265	0.0024435	0.0068755	0	0.0024816	0.0012655	0.0010181	0.0008814	0.0010602	0.0013291
68	Hohehot	0.0023812	0.0019754	0.0018235	0.0024816	0	0.001128	0.0009272	0.0008124	0.0007457	0.0008693
69	Shenyang	0.0021434	0.0021817	0.0014105	0.0012655	0.001128	0	0.0052074	0.0029036	0.0007732	0.0009071
70	Changchun	0.0015184	0.0015375	0.0011099	0.0010181	0.0009272	0.0052074	0	0.006563	0.0006733	0.0007725
71	Harbin	0.0012331	0.0012457	0.0009493	0.0008814	0.0008124	0.0029036	0.006563	0	0.0006106	0.0006911
72	Shanghai	0.0010856	0.0011978	0.0012536	0.0010602	0.0007457	0.0007732	0.0006733	0.0006106	0	0.0052417
73	Nanjing	0.0013692	0.0015525	0.0016476	0.0013291	0.0008693	0.0009071	0.0007725	0.0006911	0.0052417	0
74	Hangzhou	0.0009995	0.0010938	0.0011402	0.000978	0.000704	0.0007286	0.0006391	0.0005824	0.0079017	0.0037022
75	Hefei	0.0014788	0.0016323	0.0017377	0.0013871	0.0008938	0.0009337	0.0007917	0.0007065	0.0025825	0.0050905
76	Fuzhou	0.0006805	0.0007229	0.0008294	0.00063	0.0004809	0.000543	0.0004917	0.0004574	0.001346	0.0013529
77	Nanchang	0.0010961	0.0010999	0.0012283	0.0010287	0.000594	0.0007384	0.0006467	0.0005887	0.0018818	0.0018953
78	Jinan	0.0031957	0.0044118	0.0052766	0.0029854	0.0013645	0.0014598	0.0011402	0.0009714	0.0016442	0.0023955
79	Zhengzhou	0.0023052	0.0019113	0.003855	0.0027526	0.0011661	0.0010327	0.0008618	0.0007618	0.0015914	0.0022853
80	Wuhan	0.0012965	0.0011619	0.0016754	0.0013471	0.0008368	0.0008054	0.0006975	0.0006305	0.001286	0.0012902
81	Changsha	0.0010008	0.0009186	0.0012124	0.0010333	0.000704	0.0006805	0.0006018	0.0005513	0.0013159	0.0013224
82	Guangzhou	0.0006923	0.000652	0.0007874	0.0007081	0.0005362	0.0005223	0.0004747	0.0004427	0.0008775	0.0008804
83	Nanning	0.000619	0.0005865	0.0006939	0.0006315	0.0004911	0.0004794	0.000439	0.0004115	0.0007628	0.0007651
84	Chongqing	0.000761	0.0007122	0.0008775	0.0007953	0.0006023	0.0005604	0.000506	0.0004698	0.0006313	0.0007588
85	Chengdu	0.0007778	0.0007269	0.0008999	0.0010638	0.0007446	0.0005695	0.0005133	0.0004761	0.0006756	0.0007755
86	Guiyang	0.0006255	0.0005924	0.0007021	0.0006456	0.0005123	0.0004833	0.0004423	0.0004144	0.0007736	0.0007732
87	Kunming	0.0004998	0.0004784	0.0005475	0.0006125	0.0004913	0.0004046	0.0003755	0.0003552	0.0005885	0.0005898
88	Xian	0.0013704	0.0012208	0.0017207	0.0024397	0.0012302	0.0008333	0.0007183	0.0006475	0.0010525	0.001317
89	Lanzhou	0.000877	0.0008153	0.0009933	0.0011969	0.0013883	0.0006224	0.0005559	0.0005125	0.0007269	0.0008439
90	Xining	0.0007592	0.0007106	0.0008751	0.0010293	0.0011678	0.0005594	0.0005052	0.0004691	0.0006615	0.000757
91	Yinchuan	0.0011826	0.0010731	0.0010267	0.0012069	0.0023495	0.0007621	0.0006648	0.0006037	0.0006744	0.000774
92	Urumchi	0.0004215	0.0004061	0.000455	0.0004934	0.0005231	0.0003518	0.0003295	0.0003138	0.0003896	0.0004208

Figure S5 The unitized spatial weights matrix of 29 Chinese capital cities based on power function (partial results)

2.3 Step 3: computing SCI

By means of the formula $R_c=x^TWy$ or $R_c=y^TWx$, we can calculate the SCI value (R_c) using Excel functions “mmult” and “transpose” easily. According to the number and result arrangement in the same worksheet, in any cell, say, B36, we can input a formula as follows:

“=MMULT(MMULT(TRANSPOSE(E2:E30),H64:AJ92),D2:D30)”

Or

“=MMULT(MMULT(TRANSPOSE(D2:D30),H64:AJ92),E2:E30)”

Pressing three keys **Ctrl**, **Shift** and **Enter** at the same time yields the SCI value immediately. The result is about 0.156644, which is based on PSD (see File S2 for details).

If we turn the PSD function, stdevp, into the *sample standard deviation* (SSD) function, stdev, we will have a SCI value based on SSD. In other words, inputting a formula such as “=STDEV(B2:B30)” in cell B32 to replace the formula “=STDEVP(B2:B30)” yields a SSD value about 19924.7257, inputting a formula “=STDEV(C2:C30)” in cell C32 to replace the formula

“=STDEVP(C2:C30)” yields a SSD value about 13.4596. Accordingly, the PSD-based SCI will change to the SSD-based SCI, and the result is approximately 0.151243.

	G	H	I	J	K	L	M	N	O	P	Q
32		Beijing	Tianjin	Shijiazhuang	Taiyuan	Hohehot	Shenyang	Changchun	Harbin	Shanghai	Nanjing
33	Beijing	0	0.8573734	0.7326164	0.5651874	0.4727491	0.4350436	0.3088522	0.2353427	0.1933454	0.2717317
34	Tianjin	0.8573734	0	0.6246081	0.4818629	0.4053225	0.4414426	0.3133951	0.2388043	0.225509	0.3169351
35	Shijiazhuang	0.7326164	0.6246081	0	0.7714644	0.3759391	0.2823098	0.2004213	0.1527193	0.2409598	0.33865
36	Taiyuan	0.5651874	0.4818629	0.7714644	0	0.4873058	0.2442297	0.1733869	0.1321193	0.1858919	0.2612564
37	Hohehot	0.4727491	0.4053225	0.3759391	0.4873058	0	0.2056665	0.1460096	0.111258	0.0914039	0.1284609
38	Shenyang	0.4350436	0.4414426	0.2823098	0.2442297	0.2056665	0	0.7099339	0.5409634	0.0995493	0.1399087
39	Changchun	0.3088522	0.3133951	0.2004213	0.1733869	0.1460096	0.7099339	0	0.7619912	0.0706734	0.0993259
40	Harbin	0.2353427	0.2388043	0.1527193	0.1321193	0.111258	0.5409634	0.7619912	0	0.0538525	0.0756855
41	Shanghai	0.1933454	0.225509	0.2409598	0.1858919	0.0914039	0.0995493	0.0706734	0.0538525	0	0.7115306
42	Nanjing	0.2717317	0.3169351	0.33865	0.2612564	0.1284609	0.1399087	0.0993259	0.0756855	0.7115306	0
43	Hangzhou	0.1678301	0.1957491	0.2091609	0.1613602	0.0793415	0.086412	0.0613468	0.0467457	0.7979032	0.6176316
44	Hefei	0.2992899	0.3352439	0.3582132	0.2763487	0.1358819	0.1479909	0.1050638	0.0800577	0.5011836	0.7043739
45	Fuzhou	0.0726861	0.0847776	0.1163707	0.0589157	0.0244773	0.0374245	0.0265689	0.0202453	0.2656955	0.2674921
46	Nanchang	0.1964098	0.197516	0.2340247	0.1765311	0.0496131	0.0892731	0.063378	0.0482935	0.3875148	0.3901352
47	Jinan	0.5722139	0.6674034	0.7131308	0.550155	0.2705136	0.2946203	0.2091609	0.1593788	0.3378901	0.4748779
48	Zhengzhou	0.4612102	0.3932148	0.6295384	0.5230384	0.2165722	0.1777248	0.1261729	0.0961426	0.3259609	0.4581124
49	Wuhan	0.2525996	0.2153593	0.3447911	0.2659941	0.1186142	0.1091537	0.0774919	0.0590482	0.2497782	0.250903
50	Changsha	0.1682075	0.143409	0.2295984	0.1779246	0.0793415	0.0726861	0.0516023	0.0393205	0.2577587	0.2595017
51	Guangzhou	0.0760263	0.0648179	0.1037737	0.0805086	0.035901	0.0328526	0.0233232	0.0177721	0.1309374	0.1318228
52	Nanning	0.0560118	0.0477541	0.0764545	0.0593141	0.0264498	0.0242039	0.0171832	0.0130934	0.0964672	0.0971195
53	Chongqing	0.0959269	0.0816928	0.1309374	0.1061313	0.0517184	0.0414521	0.0294282	0.0224241	0.0592475	0.0952826
54	Chengdu	0.1009002	0.0859281	0.1377258	0.1869389	0.0910964	0.0436011	0.0309539	0.0235866	0.0713113	0.1002225
55	Guiyang	0.0577365	0.0492245	0.0788086	0.0630939	0.030746	0.0249492	0.0177123	0.0134966	0.0996612	0.0995493
56	Kunming	0.0281669	0.0240143	0.0384471	0.0543386	0.0264795	0.0121715	0.008641	0.0065844	0.0482393	0.0485655
57	Xian	0.2720371	0.2319312	0.3546103	0.4813219	0.234551	0.1175531	0.0834549	0.0635919	0.1836093	0.2580483
58	Lanzhou	0.1307904	0.1121362	0.1659555	0.2252558	0.2766593	0.0568995	0.0403949	0.0307806	0.0859281	0.1207651
59	Xining	0.0953897	0.0812353	0.1302041	0.1767295	0.2170593	0.0412199	0.0292634	0.0222985	0.0674168	0.094749
60	Yinchuan	0.2212438	0.1896886	0.1759373	0.2280562	0.4679941	0.0962507	0.0683316	0.0520681	0.0709917	0.0997732
61	Urumchi	0.0145188	0.0123645	0.0198178	0.0268992	0.0330376	0.0062739	0.0044541	0.003394	0.0102612	0.0144213

Figure S6 The spatial contiguity matrix of 29 Chinese capital cities based on negative exponential function (partial results)

3 Process of calculations based on exponential function

We have more than one way to generate a spatial contiguity matrix (Chen, 2012). In step 2 of Section 2, the power function can be substituted with an exponential function to define a distance-decay function. The standard form of a negative exponential function is as below:

$$v_{ij}=\begin{cases}\exp(-2r_{ij}/\bar{r}), & i\neq j\\ 0, & i=j\end{cases}, \tag{3}$$

where $\bar{r}$ denotes the average distance, or the mean value of the elements in the distance matrix. In cell H33, input a formula “=IF(H2=0,0,EXP(-2*H2/(SUM($H$2:$AJ$30)/(29*30))))”, strike **Enter** key, it will yield a number 0. Seizing the bottom right corner of cell H33 and dragging it

right and down produce all the spatial contiguity values based on the negative exponential function, which are shown in Figure S6. Then, summate the spatial contiguity values using equation (3). In cell AJ62, input a formula “=SUM(H33:AJ61)”, press **Enter**, yield a sum about 154.0418. Finally, transform the spatial contiguity matrix into a normalized spatial weights matrix, which are shown in Figure S7. The other and rest steps are the same as those illustrated in Section 2 (see File S2 for details).

	G	H	I	J	K	L	M	N	O	P	Q
63		Beijing	Tianjin	Shijiazhuang	Taiyuan	Hohehot	Shenyang	Changchun	Harbin	Shanghai	Nanjing
64	Beijing	0	0.0055658	0.004756	0.0036691	0.003069	0.0028242	0.002005	0.0015278	0.0012551	0.001764
65	Tianjin	0.0055658	0	0.0040548	0.0031281	0.0026312	0.0028657	0.0020345	0.0015503	0.0014639	0.0020575
66	Shijiazhuang	0.004756	0.0040548	0	0.0050081	0.0024405	0.0018327	0.0013011	0.0009914	0.0015642	0.0021984
67	Taiyuan	0.0036691	0.0031281	0.0050081	0	0.0031635	0.0015855	0.0011256	0.0008577	0.0012068	0.001696
68	Hohehot	0.003069	0.0026312	0.0024405	0.0031635	0	0.0013351	0.0009479	0.0007223	0.0005934	0.0008339
69	Shenyang	0.0028242	0.0028657	0.0018327	0.0015855	0.0013351	0	0.0046087	0.0035118	0.0006462	0.0009083
70	Changchun	0.002005	0.0020345	0.0013011	0.0011256	0.0009479	0.0046087	0	0.0049467	0.0004588	0.0006448
71	Harbin	0.0015278	0.0015503	0.0009914	0.0008577	0.0007223	0.0035118	0.0049467	0	0.0003496	0.0004913
72	Shanghai	0.0012551	0.0014639	0.0015642	0.0012068	0.0005934	0.0006462	0.0004588	0.0003496	0	0.0046191
73	Nanjing	0.001764	0.0020575	0.0021984	0.001696	0.0008339	0.0009083	0.0006448	0.0004913	0.0046191	0
74	Hangzhou	0.0010895	0.0012708	0.0013578	0.0010475	0.0005151	0.000561	0.0003982	0.0003035	0.0051798	0.0040095
75	Hefei	0.0019429	0.0021763	0.0023254	0.001794	0.0008821	0.0009607	0.000682	0.0005197	0.0032536	0.0045726
76	Fuzhou	0.0004719	0.0005504	0.0007554	0.0003825	0.0001589	0.000243	0.0001725	0.0001314	0.0017248	0.0017365
77	Nanchang	0.001275	0.0012822	0.0015192	0.001146	0.0003221	0.0005795	0.0004114	0.0003135	0.0025156	0.0025327
78	Jinan	0.0037147	0.0043326	0.0046295	0.0035715	0.0017561	0.0019126	0.0013578	0.0010346	0.0021935	0.0030828
79	Zhengzhou	0.0029941	0.0025526	0.0040868	0.0033954	0.0014059	0.0011537	0.0008191	0.0006241	0.0021161	0.0029739
80	Wuhan	0.0016398	0.0013981	0.0022383	0.0017268	0.00077	0.0007086	0.0005031	0.0003833	0.0016215	0.0016288
81	Changsha	0.001092	0.000931	0.0014905	0.001155	0.0005151	0.0004719	0.000335	0.0002553	0.0016733	0.0016846
82	Guangzhou	0.0004935	0.0004208	0.0006737	0.0005226	0.0002331	0.0002133	0.0001514	0.0001154	0.00085	0.0008558
83	Nanning	0.0003636	0.00031	0.0004963	0.0003851	0.0001717	0.0001571	0.0001115	8.5E-05	0.0006262	0.0006305
84	Chongqing	0.0006227	0.0005303	0.00085	0.000689	0.0003357	0.0002691	0.000191	0.0001456	0.0003846	0.0006186
85	Chengdu	0.000655	0.0005578	0.0008941	0.0012136	0.0005914	0.000283	0.0002009	0.0001531	0.0004629	0.0006506
86	Guiyang	0.0003748	0.0003196	0.0005116	0.0004096	0.0001996	0.000162	0.000115	8.762E-05	0.000647	0.0006462
87	Kunming	0.0001829	0.0001559	0.0002496	0.0003528	0.0001719	7.901E-05	5.61E-05	4.274E-05	0.0003132	0.0003153
88	Xian	0.001766	0.0015056	0.002302	0.0031246	0.0015226	0.0007631	0.0005418	0.0004128	0.0011919	0.0016752
89	Lanzhou	0.0008491	0.000728	0.0010773	0.0014623	0.001796	0.0003694	0.0002622	0.0001998	0.0005578	0.000784
90	Xining	0.0006192	0.0005274	0.0008453	0.0011473	0.0014091	0.0002676	0.00019	0.0001448	0.0004377	0.0006151
91	Yinchuan	0.0014363	0.0012314	0.0011421	0.0014805	0.0030381	0.0006248	0.0004436	0.000338	0.0004609	0.0006477
92	Urumchi	9.425E-05	8.027E-05	0.0001287	0.0001746	0.0002145	4.073E-05	2.891E-05	2.203E-05	6.661E-05	9.362E-05

Figure S7 The unitized spatial weights matrix of 29 Chinese capital cities based on negative exponential function (partial results)

References

Chen YG (2012). On the four types of weight functions for spatial contiguity matrix. *Letters in Spatial and Resource Sciences*, 5(2): 65-72

Chen YG (2013). New approaches for calculating Moran’s index of spatial autocorrelation. *PLoS ONE*, 8(7): e68336
